# Editorial: Exploration of the non-invasive brain-computer interface and neurorehabilitation

**DOI:** 10.3389/fnins.2024.1377665

**Published:** 2024-02-13

**Authors:** Shugeng Chen, Lin Yao, Lei Cao, Marco Caimmi, Jie Jia

**Affiliations:** ^1^Department of Rehabilitation Medicine, Huashan Hospital, Fudan University, Shanghai, China; ^2^Department of Neurobiology, Affiliated Mental Health Center and Hangzhou Seventh People's Hospital, Zhejiang University School of Medicine, Hangzhou, China; ^3^The Nanhu Brain-Computer Interface Institute, Hangzhou, China; ^4^MOE Frontiers Science Center for Brain and Brain-Machine Integration, Zhejiang University, Hangzhou, China; ^5^College of Computer Science, College of Biomedical Engineering and Instrument Science, Zhejiang University, Hangzhou, China; ^6^Department of Artificial Intelligence, Shanghai Maritime University, Shanghai, China; ^7^National Research Council (CNR), Rome, Italy; ^8^National Clinical Research Center for Aging and Medicine, Huashan Hospital, Fudan University, Shanghai, China; ^9^National Center for Neurological Disorders, Shanghai, China

**Keywords:** brain-computer interface (BCI), electroencephalogram (EEG), stroke, rehabilitation, algorithm

The non-invasive brain–computer interface (BCI) is being increasingly explored in many fields all over the world. With the development of engineering technology and rehabilitation science, non-invasive BCI is beginning to be applied in neurorehabilitation in diseases such as stroke and other neurological dysfunctions in order to reach a higher clinical efficacy. To make this progress, engineers, rehabilitation physicians, and physiotherapists, etc., are performing lots of studies to develop this application.

Our Research Topic focuses on promoting the BCI algorithm, developing an optimal BCI experimental paradigm, and exploring brain plasticity during recovery in stroke patients or those with other nervous system diseases. After being carefully and strictly reviewed, eight articles were eventually accepted. These articles presented new methodologies for non-invasive BCI rehabilitation that allow individuals to control external devices or communicate using brain signals without the need for surgery or implantation. The algorithms are novel and efficient for signal transmitting, and they involve using various techniques to aid the recovery of individuals with neurological disorders or injuries.

By combining BCI technology with a motorized hand prothesis for functional hand neurorehabilitation, Fu et al. proposed a functional-oriented portable BCI equipment and explored its efficiency for hand motor recovery after a stroke, which showed a clinically improved rehabilitation outcome and also improved BCI classification accuracy. In this closed-loop BCI system, the BCI decoding accuracy, calibration time required, and simplicity of the system (such as number of the electrodes) play an important role in a practical clinical application. In this article collection, several works are intended to solve those issues. To further enhance the decoding accuracy, especially the motor decoding, Zhang et al. put forward a local and global convolution neural network combined with a self-attention transformer to improve the classification accuracy. Moreover, the convolutional sliding window attention network was demonstrated to have a comparable classification accuracy by Huang et al.. Compared with the traditional machine learning algorithm, the deep learning approach would automatically extract rich spatial–spectrum–temporal features and demonstrated general higher offline accuracy. However, more online experiments should be further designed to demonstrate its general capability in real-time motor control. To further find the electroencephalogram (EEG) channels required, Liu and Ye have provided a way for channel selection by a domain knowledge-assisted multi-objective evolutionary algorithm. The proposed two-objective optimization model enabled the selection of a minimal number of channels without compromising classification accuracy. Moreover, to further reduce the preparation time, Luo proposed dual selections-based knowledge transfer learning for classification in a cross-subject scenario, which demonstrated its feasibility and effectiveness under multi-source to single-target and single-source to single-target cross-subject strategies on benchmark MI datasets. Moreover, Qin et al. put forward multimodal approaches by combining multiple modalities, such as combining EEG with other neuroimaging techniques or peripheral sensors, to enhance the accuracy and versatility of BCI systems. In general, more efforts are needed in the non-invasive BCI field, especially in the development of the algorithms and their testing in the online BCI paradigm, the development of the new BCI paradigm, and filling the gap between laboratory experiments and clinical usage.

As for the explorations in the subclinical and clinical efficacy in stroke rehabilitation by applying non-invasive BCI training, EEG has been a primary modality, allowing for the monitoring of brain activity through electrodes placed on the scalp. Rehabilitation programs using BCIs have shifted toward a functional-oriented approach by Fu et al. This involves designing BCI applications that target specific functional tasks related to motor skills, such as hand movements or limb control. A notable trend is the development of portable and user-friendly BCI systems. This allows individuals to engage in rehabilitation activities outside clinical settings, promoting more frequent and personalized training. Furthermore, there has been a growing emphasis on conducting rigorous clinical trials to assess the efficacy of BCI rehabilitation interventions by not only the clinical scales scores but also with brain function change indexes. Yue et al. reported significant motor performance improvements in the Action Research Arm Test (ARAT) and upper extremity Fugl-Meyer Assessment (FMA) in 16 chronic stroke patients after a 20-session action observation-driven BCI robotic training. They suggested that ipsilesional oscillation changes in the delta and beta bands may be potential biomarkers for an effective BCI intervention for chronic stroke patients. Lin et al. enrolled 15 stroke patients at the subacute or chronic stage and investigated the changes in brain connectivity and network. They found that subacute stroke patients had higher transfer efficiency of the entire brain network and weak local nodal effects, while chronic patients were the opposite. These results may help to understand the recovery pattern of the brain function in different post-stroke stages and can be used in BCI system design and EEG data analysis.

In conclusion, we expect that this Research Topic can bring references and ideas for BCI-related researchers. Non-invasive BCI and neurorehabilitation are hot topics involving engineering, medicine, and even financial investment. Advancements in machine learning and artificial intelligence have facilitated the development of adaptive BCI systems. These systems can learn and adapt to the user's abilities, providing personalized and optimized rehabilitation exercises. In the future, more BCI research related to BCI algorithm renewal and innovation, hardware development and upgrade, BCI prototype manufacture, and multi-center randomized controlled clinical studies will appear rapidly. We hope that non-invasive BCI will bring new breakthroughs in neural rehabilitation.

## Author contributions

SC: Writing—original draft, Writing—review & editing. LY: Writing—original draft, Writing—review & editing. LC: Writing—original draft, Writing—review & editing. MC: Writing—original draft, Writing—review & editing. JJ: Writing—original draft, Writing—review & editing.

